# The role of venous valves in pressure shielding

**DOI:** 10.1186/1475-925X-7-8

**Published:** 2008-02-15

**Authors:** Constantinos Zervides, Andrew J Narracott, Patricia V Lawford, David R Hose

**Affiliations:** 1 Academic Unit of Medical Physics, School of Medicine and Biomedical Sciences, The University of Sheffield, Sheffield, UK

## Abstract

**Background:**

It is widely accepted that venous valves play an important role in reducing the pressure applied to the veins under dynamic load conditions, such as the act of standing up. This understanding is, however, qualitative and not quantitative. The purpose of this paper is to quantify the pressure shielding effect and its variation with a number of system parameters.

**Methods:**

A one-dimensional mathematical model of a collapsible tube, with the facility to introduce valves at any position, was used. The model has been exercised to compute transient pressure and flow distributions along the vein under the action of an imposed gravity field (standing up).

**Results:**

A quantitative evaluation of the effect of a valve, or valves, on the shielding of the vein from peak transient pressure effects was undertaken. The model used reported that a valve decreased the dynamic pressures applied to a vein when gravity is applied by a considerable amount.

**Conclusion:**

The model has the potential to increase understanding of dynamic physical effects in venous physiology, and ultimately might be used as part of an interventional planning tool.

## Background

The motivation behind this study was a desire to understand the physiological effects of compression cuff therapy for prevention of deep vein thrombosis. It is generally accepted [1-4], that deep vein thrombosis is associated with flow stasis, particularly in and around the venous valves and their sinuses. From a survey of the literature, it rapidly became apparent that the role and quantitative performance of venous valves, even in the normal physiological state is poorly understood. Texts on venous physiology always identify the role of the valves as the control of reverse flow [4-7]; most often in the context of muscle pump action to maintain flow in the direction of the heart and sometimes in the context of postural changes and of exercise. The purpose of this paper is to explore the effects of gravity on the pressure and flow distribution in a simple representation of a vein in the leg, and in particular to quantify the role of the valves in pressure shielding under the action of standing. The effects of a range of parameters on the shielding performance of the valves are examined. It is demonstrated that the effects depend not only on the distribution, location and performance of the valves themselves, but also on the geometric and mechanical characteristics of the veins. It is anticipated that this information will be of direct interest to the vascular surgeon because it provides an indication of the likely effect of interventions, including removal or repair of valves as well as insertion of bypass grafts, on peak pressure distributions in the peripheral vasculature.

A person who stands, inactive, for a period of time will be subjected to the full hydrostatic pressure gradient in the venous system and the pressure in the veins in the foot will reach something of the order of 100 mmHg [8,9,7]. This is confirmed by Pollack [10] and by Arnoldi [11]. The presence of a valve or valves cannot shield against this static pressure – which will be associated with the physiological phenomenon of blood pooling, and related to oedema through the Starling equation [7], but it can alleviate the transient maximum that will occur as posture is changed. In the absence of valves, a simple analysis would suggest that the transient pressure peak experienced in the vein might be double the final standing pressure. Neglecting inertial and viscoelastic effects in the vessel wall, the stress in the wall of the vein is proportional to the instantaneous applied pressure, and it is postulated that incompetent valves do not provide adequate transient pressure shielding and thus might be strongly implicated in the formation of varicosities [9,12,13]. A primary quantitative measure of valve, or rather system, performance is given by its effectiveness in reducing the pressure peaks associated with the transient response. A second measure might be one of the effectiveness of a postural change, and the associated action of gravity on valve opening and closure characteristics and thus on the 'wash out' of the sinuses and displacement of stationary pockets of blood. Although a full three-dimensional analysis is required to address this question in detail, the one-dimensional model presented in this paper is used to examine whether the properties of the system are such that there might be sufficient backflow to close the valve for realistic geometries.

## Materials and Methods

**Figure 1:**
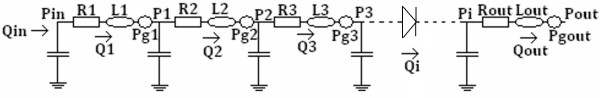
**Schematic representation of tube model.** The resistive component represents the viscous resistance of the blood, the inductive component the inertia of the blood, and the capacitive component the elasticity of the vein. A perfect valve is represented by a diode.

The methodology adopted in this study involves the construction and analysis of a numerical model that is able to capture the spatial and temporal pressure distributions in a collapsible tube that includes valves. The vein is represented as an analogous electrical circuit as illustrated in Figure 1. For simplicity, and because the focus of this study is to capture the characteristic effect of the valves, the vein is represented as a straight collapsible tube. The chosen geometrical configuration is representative of a vein from the lower extremities. The resistive component represents the viscous resistance of the blood, the inductive component the inertia of the blood, and the capacitive component the elasticity (and thus storage capacity) of the vein. A perfect valve is assumed to allow no backflow, and can be represented as a diode (in the analogous electric circuit a diode allows current to flow in one direction and blocks it from flowing in the opposite direction). A real valve will permit some backflow, associated partly with the swept volume of the valve leaflets during the closure phase, and this is represented by allowing a finite volume of blood to pass through the valve before closure. A 'leaky' valve is assumed to allow a fixed rate of leakage, although this could readily be modified to make the leakage proportional to the pressure drop across the valve segment.

A series of ordinary differential equations are written to represent the electrical system. The nonlinear elastic properties of the vein, including those associated with collapse, are represented by a tube law [14-17]. The main purpose of the tube law is to capture the vein's flexibility at small negative pressures as collapse is initiated, whilst maintaining the properties of a stiffening response for higher negative or positive pressures. A penalty of this formulation is that it does not reduce to the standard linear approximation at small positive pressures, and for the current work, a modification has been implemented to remedy this deficiency. A number of numerical techniques are available for solution of the derived equations [18]. The one adopted for the current study is a Lax Wendroff formulation, which is accurate to second order in time and space. For completeness, the governing equations and the numerical discretisation are listed in Appendix 1. This formulation has been adopted in other studies of the cardiovascular system [19], although Brook [16,20], has identified conditions under which numerical instabilities might be manifest. Numerical testing has indicated that the system is stable under the pertinent conditions for the current study.

One of the important properties of the system that will have significant influence on the results is the boundary conditions applied at the proximal and distal ends of the vein segment. For the purposes of the current study, a constant atmospheric pressure boundary condition has been applied at the proximal end and a constant flow boundary condition at the distal end. It is recognised that the prescribed boundary conditions might represent a gross simplification of physiological flow in the venous segments of interest. The important feature of the proximal pressure boundary condition is that it allows unimpeded reverse flow into the vein segment as gravity acts. It would be possible to apply a negative pressure representative of that in the thoracic cavity, but as a constant offset this would not significantly affect the results. A transient thoracic pressure representative of respiration could also be applied, but primary focus in this paper is on relatively short term events associated with a near-instantaneous application of gravity. The relatively low frequency respiratory cycle would not significantly modify the results. It has further been assumed that there is a constant flow into the 'bottom' (distal end) of the vein, based on average steady state drainage into the femoral vein. It is unlikely that there will be significant backflow through the distal end during gravity application, due to the higher resistance of the smaller vessels. More sophisticated descriptions of transient flow waveforms measured under a range of conditions can be found in the literature. Of most direct interest is the study reported by Raju S et al [21], who describe flow conditions under ambulatory conditions but not under first application of the gravity field, whilst Neglen and Raju [22] also focus on the measurement of ambulatory pressures in individuals with signs of chronic venous deficiency. Willeput R et al [23] and Abu-Yousef M [24] focus on rest and respiratory conditions. Again, it is suggested that the frequencies associated with these temporal variations are relatively low compared with those associated with the phenomenon addressed in this paper. Furthermore, the starting condition for the analysis is a steady flow through the system (equal to the distal end flow), with no gravity applied.

This paper focuses on the transient pressure and flow distributions in a vein segment, with and without valves, under a near-instantaneous application of gravity. The system is considered passive, and effects of the muscle pump are not included: similarly other relatively low frequency external load factors are neglected. A body force is applied, in the opposite direction to flow, representing the action of gravity under a change of posture from horizontal to vertical: this force is sigmoidal in time, so that there is smooth transition from zero to the full gravity force, which is then held constant.

## Results and Discussion

### Baseline condition, no valve

A series of numerical tests were performed, to ensure that the model performed properly and returned accurate results for simple conditions, including for example using a linear tube law, for which analytical comparisons were available, and for other conditions for which numerical results have been published [17,19]. Once these tests were passed, a first baseline analysis was performed using the following parameters: vein diameter 1.19 cm [25], vein thickness-to-diameter ratio 0.2 [26], vein length 1 m, wall stiffness 1 MPa [27], blood viscosity 0.004 Pa.s, blood density 1000 kg/m^3^, distal (inlet) flow 15.1 ml/s [25], proximal (outlet) pressure 0 mmHg (0 Pa), near instantaneous body-force application (gravity increased from zero to 9.8 m/s^2 ^over 0.01 milliseconds). These values are given in convenient units: all analyses were performed in SI units. The initial condition, prior to the application of gravity, was a steady flow in the opposite direction to that in which gravity would be applied (i.e from distal to proximal end of the tube).

Discretisation-independence tests were performed to ensure that the results did not depend either on the number of elements used to represent the geometry of the vessel or on the simulation time-step. Results for the baseline condition and for several parameter variations are reported in Table 1. One of the most important results is the 'dynamic pressure ratio'. This is defined as the ratio of the peak dynamic pressure to the unavoidable static pressure that will be reached when the system has stabilised. Also reported in Table 1 are the magnitudes of the first and second pressure peaks recorded as the system oscillates (to give an indication of how quickly the overall peak is reached), the time for which the valve remains closed during the first oscillatory phase, and measures of the peak positive and negative area changes as the vein expands and collapses.

**Table 1:** Baseline condition, valve performance and gravity application time results

**Parameters**	**No valve**	**Perfect valve**	**"Real" valve**	**Leaky valve**	**Gravity test**
**Diameter (cm)**	1.2	1.2	1.2	1.2	1.2
**Length (m)**	1	1	1	1	1
**Young's modulus (kPa)**	1000	1000	1000	1000	1000
**Poisson's ratio**	0.5	0.5	0.5	0.5	0.5
**Blood viscosity (mPas)**	4	4	4	4	4
**Blood density (Kgm**^-3^**)**	1000	1000	1000	1000	1000
**Gravity application time (s)**	Near instantaneous	Near instantaneous	Near instantaneous	Near instantaneous	0.1
**Valve distribution**	No valve	One	One	One	One
**Valve location from inlet (m)**	No valve	0.5	0.5	0.5	0.5
**Valve performance**	No valve	Perfect	"Real"	Leaky	Perfect
**First peak (mmHg)**	136.9 (18.2 kPa)	60.2 (8.01 kPa)	60.3 (8.02 kPa)	65.4 (8.69 kPa)	50.3 (6.69 kPa)
**Second peak (mmHg)**	127.1 (16.9 kPa)	81.9 (10.9 kPa)	81.9 (10.9 kPa)	115.8 (15.4 kPa)	72.6 (9.66 kPa)
**Maximum pressure (mmHg)**	136.9 (18.2 kPa)	93.3 (12.4 kPa)	93.9 (12.4 kPa)	115.8 (15.4 kPa)	99.3 (13.2 kPa)
**Dynamic pressure ratio**	1.83	1.25	1.25	1.51	1.33
**Valve closed time (s)**	No valve	0.17	0.17	0.11	0.16
**Maximum collapse (%)**	0.02	20.2	20.4	3.57	8.17
**Maximum expansion (%)**	15.1	9.89	9.89	12.5	10.6

**Figure 2:**
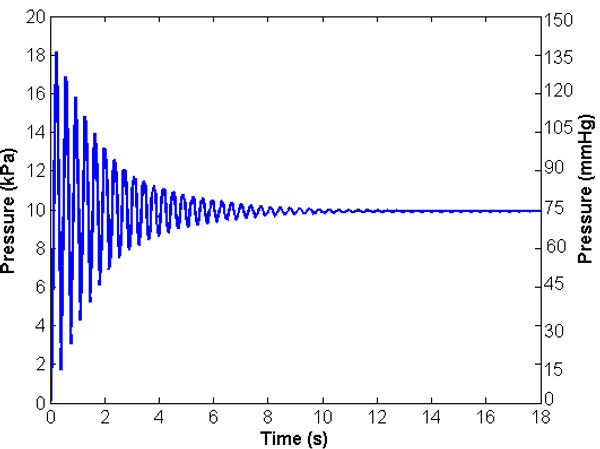
**Baseline condition pressure against time at the distal end of the vessel.** The system is oscillatory but after approximately 12 s the pressure at the distal end remains within 2% of the steady state value at 74.5 mmHg (9936.3 Pa), consistent with the hydrostatic force applied plus the (small) pressure drop associated with the superimposed steady flow.

Figure 2 presents the computed pressure against time at the distal end of the vessel. The system is oscillatory (the only damping in this system is that due to the viscosity of the blood – it is recognised that the real system will have additional damping due to the viscoelastic properties of the vessel wall, and probably more importantly of the surrounding tissues), but after a period of approximately 12 s the pressure at the distal end remains within 2% of the steady state value at 74.5 mmHg (9936.3 Pa), consistent with the hydrostatic force applied plus the (small) pressure drop associated with the superimposed steady flow. The overshoot associated with the dynamic system produces a peak pressure of 136.5 mmHg (18151 Pa), representing a dynamic pressure ratio of 1.83 (a simple first principles analysis without damping would suggest a ratio of 2.00 [28], so this result is plausible).

**Figure 3:**
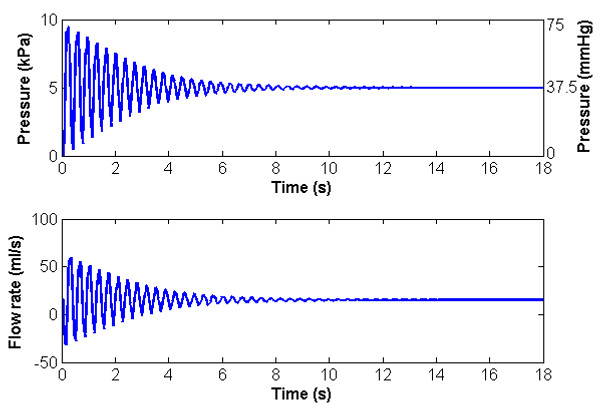
**Baseline condition pressure against time and flow rate against time at the midpoint of the vessel.** The pressure exhibits similar characteristics to that at the distal end, oscillating about its hydrostatic condition of one-half of the distal end value. The flow starts from the initial condition, oscillates in response to the sudden application of gravity, and returns to the steady condition after approximately 12 s.

Figure 3 illustrates the pressure and flow against time at a point halfway along the vein. The pressure exhibits similar characteristics to that at the distal end, oscillating about its hydrostatic condition of one-half of the distal end value. The flow starts from the initial condition, oscillates in response to the sudden application of gravity, and returns to the steady condition after approximately 12 s. It is noted that there is very significant reverse flow (≈30 ml/s) occurring approximately 80 ms after gravity is applied. The reverse flow acts to fill the more distal sections of the vein as it distends under the increased (gravitational) pressure. As demonstrated later, this reverse flow would be enough to close a competent valve and consequently to shield the lower parts of the vein and reduce peak dynamic pressures. If there were sufficient inflow from below (unlikely in the human system unless the process of standing is done very slowly) then the vein could be filled (distended) entirely by the inflow, and reverse flow might not occur.

The analysis of the baseline condition gives some confidence in the operation of the model, and also provides quantitative information on the peak pressure that can be expected at the distal end of the vein in the absence of protection from venous valves. Further confidence has been developed by comparison of the results with those from three dimensional models using a commercial finite element code, but the reporting of these results is beyond the scope of this paper.

### Effect of perfect, real and incompetent valves

The model was next used to evaluate and to provide quantitative information about a hypothesis often expressed in text book descriptions of venous physiology, e.g. Browse [13]:

'The venous valves normally protect the wall of the vein below each valve from the pressure in the vein above it.'

**Figure 4:**
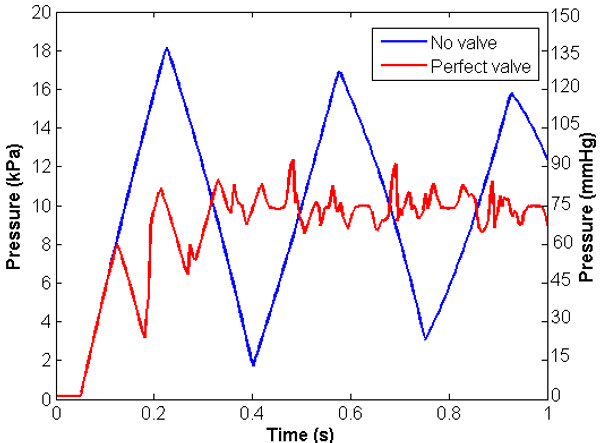
**Pressure against time at the distal end of the vessel with and without perfect valve.** The presence of the valve significantly changes the form of the dynamic response and the approach towards the steady state is reasonably asymptotic, with relatively lower pressure oscillations superimposed on the asymptote.

A perfect (no reverse flow) valve was introduced into the vein at a point halfway along its length, and a simulation performed to illustrate the effect of the valve on the peak pressures and flows in the system. The oscillatory nature of the system is such that the valve will open and close several times before finally settling in the open state in the hydrostatic condition. Attention is focused on the early phase, from the time of gravity application through to the time of peak pressure in the system. Figure 4 illustrates the pressure at the distal end against time during the first second after application of gravity, together with the results for the case with no valve. The distinctive saw-tooth appearance of the oscillations is due to the summation of pressure waves as they reflect from the domain boundaries. The presence of the valve significantly changes the form of the dynamic response. Without the valve, the system undergoes relatively high-amplitude oscillations about the hydrostatic pressure value, gradually damping towards the steady state. With the valve, the approach towards the steady state is reasonably asymptotic, with relatively lower pressure oscillations superimposed on the asymptote. The peak pressure for the system with a valve is 93.0 mmHg (12370 Pa), representing a dynamic pressure ratio of 1.25. Perhaps, therefore, the most important observation is that, consistent with the hypothesis, the valve has provided a very significant shielding effect (over 45 mmHg (5985 Pa) reduction in peak pressure).

**Figure 5:**
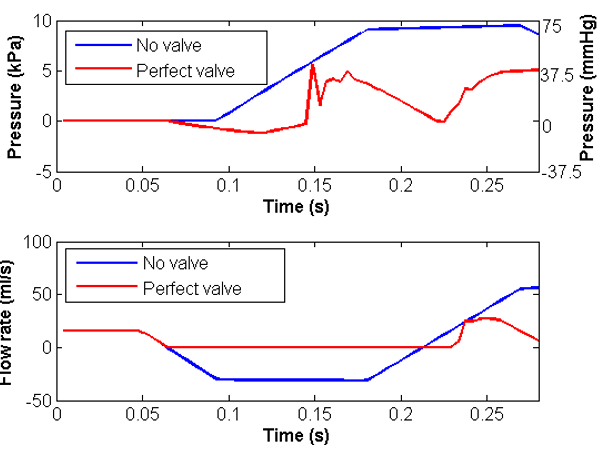
**Pressure against time and flow rate against time at the midpoint of the vessel with and without a perfect valve.** Here the effect of the valve is very clearly indicated since the analyses are identical up to the point at which flow reversal occurs.

**Figure 6:**
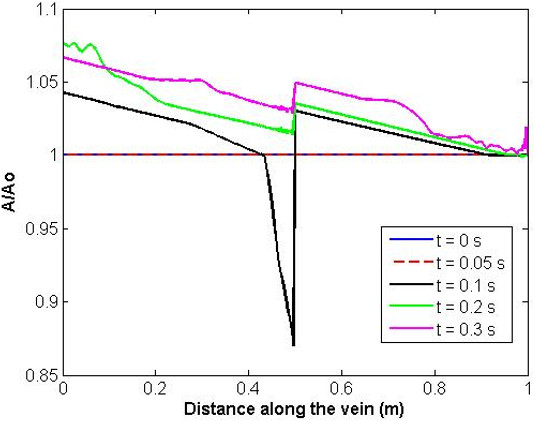
Ratio of cross-sectional area over undeformed cross sectional area along the vein length for increasing time.

**Figure 7:**
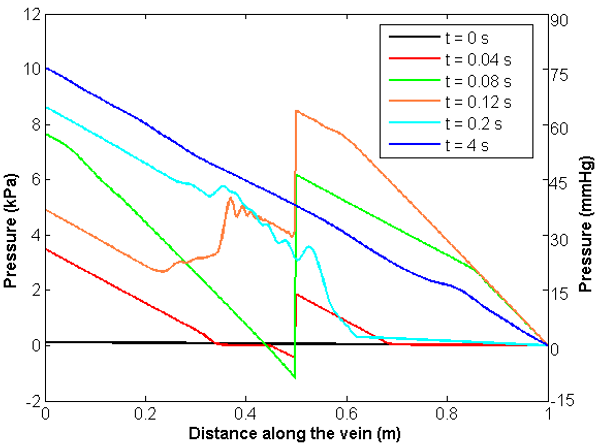
Pressure along the vein length for increasing time showing a pressure discontinuity at the valve location.

Figure 5 illustrates pressure and flow at the section immediately distal to the valve, together with the no-valve system. Here the effect of the valve is very clearly indicated. The analyses are identical up to the point at which flow reversal occurs. At this point, the valve closes and the flow is set to zero until forward flow is re-established, partly by the constant influx from the distal boundary and partly by wave reflection. The segment of vein distal to the valve now acts as a closed cylinder (at least in the portion immediately distal to the valve which takes some time to be affected by the constant influx from the (relatively distant) distal boundary). Blood continues to fall towards the distal end but at a significantly reduced rate, and the segment immediately distal to the valve reduces in diameter and starts to collapse. The degree of collapse is determined by the rate of application of gravity and the physical characteristics of the system. In the model reported here, the area reduction immediately distal to the valve is approximately 20%. Figure 6 illustrates the ratio of cross-sectional area to undeformed cross-sectional area along the length of the vein at different points in time and Figure 7 illustrates the pressure variation along the length of the vein at different points in time, showing clearly the pressure discontinuity at the valve. The presence of waves reflecting from proximal and distal boundaries is also apparent. After approximately 6.3 s, the system settles to within 2% of the hydrostatic state.

**Figure 8:**
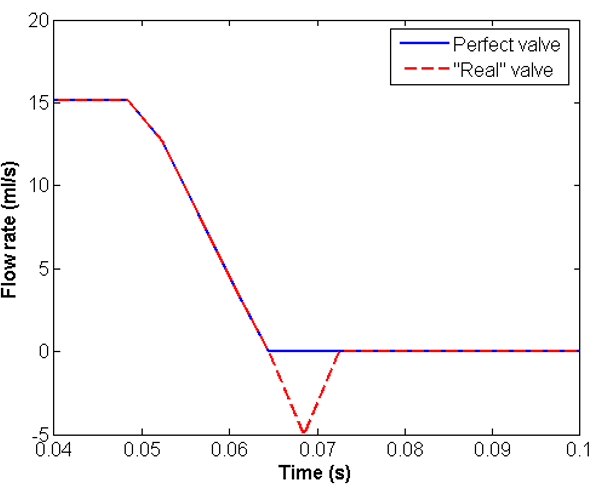
Flow rate vs time through a perfect and "real" valve.

The above results illustrate the effect of a perfect valve on pressure shielding. A real valve must permit some reverse flow as it is swept to the closed position. A first estimate of the volume of flow reversal (neglecting the reverse flow as the valve is first entrained) can be made by measuring the swept volume of the valve during closure. An estimate of this volume, based on conic sections is 0.12 ml. Results based on this approximation are illustrated in Figure 8, together with those for the perfect valve. Although the 'real' valve allows some regurgitation as the leaflets are swept to closure, the volume associated with this event is small and the effect is negligible; this might not be true if a larger regurgitant volume were to be admitted, reflecting the entrainment of the leaflets in the reversing flow. An incompetent valve might be expected to lie further towards the no-valve condition. To test this hypothesis, an analysis has been performed in which the negative flow rate through the valve has been limited to 20 ml/s. In this condition, the pressure shielding effect is substantially reduced, and the dynamic pressure ratio is increased (from 1.25 for the perfect valve) to 1.51.

In a final test on the reference configuration with a perfect valve, the time over which gravity was applied was increased from near instantaneous to 100 ms (more consistent with the likely time taken to stand up). As expected, the first and second pressure peaks were lower (by the order of 10%) but perhaps surprisingly, the absolute peak was a little higher. This was due to different interactions of the wave reflections in the system, but it does not affect the overall shape of the response, nor indeed the conclusions.

### Parameter studies

The model permits the examination of the effect of change of the geometrical and mechanical characteristics of the system on the pressure-shielding phenomenon. Results for variations of a number of parameters are presented in Table 2. Reducing the undeformed diameter of the vein by one-third increases the dynamic pressure ratio to 1.47 in the presence of the valve, and increasing the diameter of the vein by one third reduces the peak pressure ratio to 1.22. Similarly, doubling the vein stiffness causes an increase to 1.41, and halving it causes a decrease to 1.21. Each of these results is qualitatively consistent with expectations based on an understanding of the physics phenomena: the model provides quantification of the effect.

**Table 2:** Parameter variation test results

**Parameters**	**Perfect valve**	**Diameter test**		**Stiffness test**		**Distribution test**	**Length test**		**Location test**	
**Diameter (cm)**	1.2	0.8	1.6	1.2		1.2	1.2		1.2	
**Length (m)**	1	1		1		1	0.50	0.75	1	
**Young's modulus (kPa)**	1000	1000		500	2000	1000	1000		1000	
**Poisson's ratio**	0.5	0.5		0.5		0.5	0.5		0.5	
**Blood viscosity (mPas)**	4	4		4		4	4		4	
**Blood density (Kgm^-3^)**	1000	1000		1000		1000	1000		1000	
**Gravity application time (s)**	Near instantaneous	Near instantaneous		Near instantaneous		Near instantaneous	Near instantaneous		Near instantaneous	
**Valve distribution**	One	One		One		Two	One		One	
**Valve location from inlet (m)**	0.5	0.5		0.5		0.25 and 0.75	0.5		0.25	0.75
**Valve performance**	Perfect	Perfect		Perfect		Perfect	Perfect		Perfect	
**First peak (mmHg)**	60.2 (8.01 kPa)	66.3 (8.81 kPa)	56.8 (7.55 kPa)	56.3 (7.48 kPa)	63.2 (8.4 kPa)	32.6 (4.34 kPa)	33.2 (4.41 kPa)	46.8 (6.23 kPa)	32.6 (4.33 kPa)	87.2 (11.6 kPa)
**Second peak (mmHg)**	81.9 (10.9 kPa)	114.3 (15.2 kPa)	74.9 (9.97 kPa)	74.6 (9.92 kPa)	95.5 (12.7 kPa)	55.6 (7.39 kPa)	55.9 (7.44 kPa)	57 (7.58 kPa)	99.3 (13.2 kPa)	90.2 (12 kPa)
**Maximum pressure (mmHg)**	93.3 (12.4 kPa)	115.8 (15.4 kPa)	89.5 (11.9 kPa)	90.9 (12.1 kPa)	104.5 (13.9 kPa)	92.9 (12.36 kPa)	57.2 (7.61 kPa)	77.5 (10.31 kPa)	110.5 (14.7 kPa)	105.3 (14 kPa)
**Dynamic pressure ratio**	1.25	1.47	1.22	1.21	1.41	1.25	1.53	1.39	1.49	1.41
**Valve closed time (s)**	0.17	0.11	0.19	0.26	0.11	0.09 and 0.17	0.06	0.11	0.16	0.15
**Maximum collapse (%)**	20.2	3.64	24.5	48.2	6.06	35.6	3.58	10.6	3.25	42.3
**Maximum expansion (%)**	9.89	12.1	9.87	21.2	5.41	9.89	5.92	7.19	11.9	11.5

Changing the overall length of the system (whilst maintaining the position of the valve at 0.5 m from the inlet), or changing the position of the valve along the length of the 1 m vein, increased the dynamic pressure ratio, suggesting that the optimal position for a valve in a vein with the imposed boundary conditions is near to the midpoint. Finally, a test with two valves, one at one-quarter length and one at three-quarters length produced a pressure shielding of the same magnitude as that obtained with a single valve halfway along the vein.

## Conclusion

The one-dimensional model reported in this paper permits the quantitative evaluation of the effects of venous valves on the loads and geometrical changes induced by the action of gravity. It is an important first step in a longer-term study of venous valves, venous diseases and their prevention. With refinements to the venous valve description, applied tube law and boundary conditions, a more physiologically realistic model can be created in an equivalent form to the Westerhof arterial model [29]. This will enable the model to be validated against physiological data. For the purposes of this paper though the greatest interest is in the dynamic pressure ratio, which provides a measure of the increase of the peak local pressure in the system (due to dynamic effects) over the corresponding hydrostatic pressure. It is demonstrated that, for a configuration typical of the femoral vein, the dynamic pressure ratio without a valve is 1.83, and that with a perfect valve located halfway along the vein is 1.25. The absolute pressure reduction is over 40 mmHg (5320 Pa). The model has been used to investigate the quantitative influence of variation of a number of parameters. Following extensive *in vitro *and *in vivo *validation, this model might be used to evaluate the effects of valve incompetence on venous pressure distributions and could have implications for the understanding of the progression of disease in the context of varicose veins. It might also be used as part of an interventional planning tool.

The reported study is entirely theoretical. Validation against other reported numerical studies has been performed to give confidence in the numerical implementation, but validation against experimental data is an important next step. Some experimental data does exist for collapsible tubes with gravity effects, for example of the filling under gravity of an initially collapsed tube [30], but none of direct relevance to the current study. A preliminary experimental model that can be used directly to validate the current model has been reported by Potter [31] and Burnett [32] but this is not yet sufficiently mature for detailed comparative evaluation. Validation against physiological data, such as that presented in the works referenced in the section on boundary conditions, will require first the construction of an improved numerical model with a more complex network representation of the venous circulation in the lower limb. In the longer term a detailed three dimensional (3D) model is required to compute the haemodynamic characteristics in the region of the valve, and to evaluate the effects of local geometric and material variations. The 1D model described in this paper provides important mutual validation data for such a 3D model, as well as the potential to provide local boundary conditions for it in the region of the valve.

## Competing interests

The author(s) declare that this work was funded by the British Heart Foundation which also financed this manuscript.

## Authors' contributions

CZ carried out all of the computational work reported in this manuscript. DRH drafted the framework of the manuscript, integrated the computational results provided by CZ and drafted the conclusions. DRH, PVL and AJN conceived the program of work, supervised its progression and provided distinct and separate intellectual input. All authors participated in the critical review and revision of this manuscript prior to submission.

## Appendix: Equations and discretisation

### A1: Governing equations

The continuity equation used was:

$$C^{\prime }\frac{\partial P}{\partial t}+\frac{\partial Q}{\partial z}=0$$

where

$${C^{\prime }}_{i}^{n}=\frac{dA^{n}}{dP_{i}}l=\frac{1}{\left(10Kp\left(\frac{{\left(A_{i}^{n}\right)}^{9}}{A_{o}^{10}}\right)+1.5Kp\left(\frac{\left(A_{o}^{1.5}\right)}{\sqrt{\left(A_{i}^{n}\right)}}\right)\right)}$$

The momentum equation used was:

$$L^{\prime }\frac{\partial Q}{\partial t}+\frac{\partial P}{\partial z}=-R^{\prime }Q$$

where

$${R^{\prime }}_{i}^{n}=\frac{8\pi \mu }{{\left(A_{i}^{n}\right)}^{2}}$$

$${L^{\prime }}_{i}^{n}=\frac{\rho }{\left(A_{i}^{n}\right)}$$

The tube law used was:

$$P_{i}^{n}-P_{e}=Kp\left({\left(\frac{A_{i}^{n}}{A_{o}}\right)}^{z}-{\left(\frac{A_{i}^{n}}{A_{o}}\right)}^{-1.5}\right)$$

where

$$Kp=\frac{E}{12\left(1-\sigma^{2}\right)}{\left(\frac{h}{r}\right)}^{3}$$

The variables used in equations 1–7 were: A_0 _– undeformed vessel area-, A^n^_i _– vessel area at point i along the vessel length at time n-, μ – fluid viscosity-, ρ – fluid density-, E – vessel wall Young's modulus, σ-vessel Poisson's ratio, h – vessel wall thickness and finally r – vessel radius-. All variables used were described in SI units.

### A2: Lax-Wendroff discretisation

To find the two equations of interest for pressure and flow using the Lax-Wendroff technique the following two equations have to be used.

$$P_{i}^{t+\Delta t}=P_{i}^{t}+\Delta t{\left(\frac{\partial P}{\partial t}\right)}_{i}+\frac{\Delta t^{2}}{2}{\left(\frac{\partial^{2}P}{\partial t^{2}}\right)}_{i}$$

$$Q_{i}^{t+\Delta t}=Q_{i}^{t}+\Delta t{\left(\frac{\partial Q}{\partial t}\right)}_{i}+\frac{\Delta t^{2}}{2}{\left(\frac{\partial^{2}Q}{\partial t^{2}}\right)}_{i}$$

The above two equations are the Taylor series expansion of second order accuracy. Based on the above two equations and the simplified version of the mass and momentum conservation equations above, the equations needed for pressure and flow using the Lax-Wendroff technique can be found. Firstly for pressure to be found the first and second time derivatives of pressure have to be found. The first one is easily found from the continuity equation and is:

$$C^{'}\frac{\partial P}{\partial t}+\frac{\partial Q}{\partial x}=0\Rightarrow \frac{\partial P}{\partial t}=-\frac{1}{C'}\frac{\partial Q}{\partial x}$$

In order to find the second time derivative of pressure the first time derivative of pressure must be differentiated in time. This gives:

$$\frac{\partial P}{\partial t}=-\frac{1}{C'}\frac{\partial Q}{\partial x}\Rightarrow \frac{\partial }{\partial t}\left(\frac{\partial P}{\partial t}\right)=-\frac{1}{C'}\frac{\partial }{\partial t}\left(\frac{\partial Q}{\partial x}\right)\Rightarrow \frac{\partial^{2}P}{\partial t^{2}}=-\frac{1}{C'}\frac{\partial^{2}Q}{\partial t\partial x}$$

Thus to find the second time derivative of pressure $\frac{\partial^{2}Q}{\partial t\partial x}$ must be found. This is done by differentiating the momentum equations in space. This gives:

$$\begin{array}{l} L^{'}\frac{\partial Q}{\partial t}+\frac{\partial P}{\partial x}=-R^{'}Q\Rightarrow \frac{\partial Q}{\partial t}=-\frac{1}{L'}\frac{\partial P}{\partial x}-\frac{R^{'}}{L'}Q\Rightarrow \frac{\partial }{\partial x}(\frac{\partial Q}{\partial t})=-\frac{1}{L'}\frac{\partial }{\partial x}\left(\frac{\partial P}{\partial x}\right)-\frac{R^{'}}{L'}\frac{\partial }{\partial x}\left(Q\right)\\ \Rightarrow \frac{\partial^{2}Q}{\partial t\partial x}=-\frac{1}{L'}\frac{\partial^{2}P}{\partial x^{2}}-\frac{R^{'}}{L'}\frac{\partial Q}{\partial x} \end{array}$$

Now by substituting 12 in 11 the second time derivative of pressure is found to be:

$$\frac{\partial^{2}P}{\partial t^{2}}=-\frac{1}{C'}\frac{\partial^{2}Q}{\partial t\partial x}\Rightarrow \frac{\partial^{2}P}{\partial t^{2}}=-\frac{1}{C'}\left[-\frac{1}{L'}\frac{\partial^{2}P}{\partial x^{2}}-\frac{R^{'}}{L'}\frac{\partial Q}{\partial x}\right]$$

Since both the first and second time derivative of pressure are found, equation 8 can be solved. This gives:

$$\begin{array}{l} P_{i}^{t+\Delta t}=P_{i}^{t}+\Delta t{\left(\frac{\partial P}{\partial t}\right)}_{i}+\frac{\Delta t^{2}}{2}{\left(\frac{\partial^{2}P}{\partial t^{2}}\right)}_{i}\Rightarrow \\ P_{i}^{t+\Delta t}=P_{i}^{t}+\Delta t\left[-\frac{1}{C'}{\frac{\partial Q}{\partial x}|}_{i}\right]+\frac{\Delta t^{2}}{2}\left[-\frac{1}{C'}\left[-\frac{1}{L'}{\frac{\partial^{2}P}{\partial x^{2}}|}_{i}-\frac{R^{'}}{L'}{\frac{\partial Q}{\partial x}|}_{i}\right]\right]\Rightarrow \\ P_{i}^{t+\Delta t}=P_{i}^{t}-\frac{\Delta t}{C'}{\frac{\partial Q}{\partial x}|}_{i}+\frac{\Delta t^{2}}{2C'L'}\left[{\frac{\partial^{2}P}{\partial x^{2}}|}_{i}\right]+\frac{\Delta t^{2}R'}{2C'L'}{\frac{\partial Q}{\partial x}|}_{i}\Rightarrow \\ P_{i}^{t+\Delta t}=P_{i}^{t}+\frac{\Delta t^{2}}{2CL}\left[P_{i+1}^{t}-2P_{i}^{t}+P_{i-1}^{t}\right]-\frac{\Delta t}{2C}\left[Q_{i+1}^{t}-Q_{i-1}^{t}\right]+\frac{\Delta t^{2}R'}{2CL'}\left[Q_{i}^{t}-Q_{i-1}^{t}\right] \end{array}$$

For flow to be found the same methodology as used to find the pressure equation is used and gives:

$$Q_{i}^{t+\Delta t}=Q_{i}^{t}\left(1-\frac{\Delta tR'}{L'}\right)-\frac{\Delta t}{2L}\left(P_{i+1}^{t}-P_{i-1}^{t}\right)+\frac{\Delta t^{2}}{2LC}\left[Q_{i+1}^{t}-2Q_{i}^{t}+Q_{i-1}^{t}\right]+\frac{\Delta t^{2}R'}{2LL'}\left(P_{i+1}^{t}-P_{i}^{t}\right)+Q_{i}^{t}\frac{\Delta t^{2}R{'}^{2}}{2L{'}^{2}}$$
